# Protocol for the evaluation of a pay for performance programme in Pwani region in Tanzania: A controlled before and after study

**DOI:** 10.1186/1748-5908-8-80

**Published:** 2013-07-19

**Authors:** Josephine Borghi, Iddy Mayumana, Irene Mashasi, Peter Binyaruka, Edith Patouillard, Ikunda Njau, Ottar Maestad, Salim Abdulla, Masuma Mamdani

**Affiliations:** 1 Department of Global Health and Developmnent, London School of Hygiene and Tropical Medicine, 15-17 Tavistock Place London WC1H NSH, UK; 2 Ifakara Health Institute, Plot 463, Kiko Avenue Mikocheni, Dar es Salaam, Tanzania; 3 CHR Michelsen Institute, Jekteviksbakken 31, Bergen, Norway

**Keywords:** Pay for performance, Impact evaluation, Process evaluation, Economic evaluation, Tanzania, Protocol

## Abstract

**Background:**

The use of supply-side incentives to increase health service utilisation and enhance service quality is gaining momentum in many low- and middle-income countries. However, there is a paucity of evidence on the impact of such schemes, their cost-effectiveness, and the process of implementation and potential unintended consequences in these settings. A pay for performance (P4P) programme was introduced in Pwani region of Tanzania in 2011.

**Methods/design:**

An evaluation of the programme will be carried out to inform a potential national rollout. A controlled before and after study will examine the effect of the P4P programme on quality, coverage, and cost of targeted maternal and newborn healthcare services and selected non-targeted services at facilities in Tanzania. Data will be collected from a survey of 75 facilities, 750 patients exiting consultations, over 75 health workers, and 1,500 households of women who delivered in the previous year, in all seven intervention districts. Data will be collected from the same number of respondents in four control districts. A process evaluation will examine: whether the P4P programme was implemented as planned; stakeholder response to the programme and its acceptability; and implementation bottlenecks and facilitating factors. Three rounds of process data collection will be conducted including a review of available P4P documents, individual interviews and focus group discussions with key informants working at facility and district level in five of the intervention districts, and at the regional and national levels. An economic evaluation will measure the cost-effectiveness of P4P relative to current practice from a societal perspective.

**Discussion:**

This evaluation will contribute robust evidence on the impact and cost-effectiveness of P4P in a low income setting, as well as generate a better understanding of the feasibility of integrating complex intervention packages like P4P within health systems in resource poor settings.

## Background

Many countries are struggling to meet the Millennium Development Goals, and achieve acceptable and effective coverage levels for specific services, especially those targeting mothers and children [1,2]. As a result, the need to identify mechanisms for stimulating higher levels of service use, and ensure adequate service quality has become paramount. There is renewed interest in the potential for incentives, and specifically financial incentives, to address these issues.

Performance based financing, payment for performance or results based financing is ‘an intervention designed to increase the quantity and quality of healthcare’ [3]. Payment for performance (P4P) programmes can be applied on the demand or the supply side. Supply-side incentives are typically targeted at health managers, health institutions, and/or their staff, and tied to the achievement of pre-defined performance indicators that are set out in a performance contract [4]. Fundamentally, the approach assumes recipients will respond to financial incentives and be motivated to produce more and better outcomes. It has further been hypothesized that such incentives can stimulate more radical system reform by increasing system responsiveness to user needs, promoting the use of health information systems, and providing greater financial autonomy to health facilities [4].

There is substantive experience with P4P mechanisms in the health sector in high income countries. A recent review of 25 systematic reviews in high income countries, concluded that performance based financing ‘… can be effective in the short run for simple and distinct, well defined behavioural goals’ [5]. There is now growing interest in the application of such schemes in low- and middle-income countries.

The current evidence from lower income countries is, however, much weaker. While many studies show promising results (*e*.*g*., [6-11]), they generally suffer from methodological weakness, with the exception of [11], notably a lack of control group, and inability to control for possible confounders, limiting the conclusiveness of evidence in relation to impact. A recent systematic review of performance based financing in low and middle income settings concluded that: ‘the current evidence base is too weak to draw general conclusions.... Longer term questions of sustainability and cost-effectiveness remain untested, and adverse consequences have not been considered in most cases’ [12].

Hence, there is considerable uncertainty, not just about the impact, but also the sustainability of P4P effects over time, and whether such programmes represent value for money in relation to other possible investments [13-16]. Furthermore, there has been limited exploration of how P4P interacts with the health system, how it is perceived by different stakeholders, and whether there may be potential for unintended consequences. However, a few recent publications have begun to address this issue by adopting an ‘open box’ approach to evaluation, which seeks to understand how interventions work, rather than to simply measure if they work (*e*.*g*., [17,18]).

The potential risks associated with P4P are multi-fold [19], including diverting health worker behaviour away from un-incentivised but equally important activities. A further risk includes supplier-induced demand, or the unnecessary/excessive provision of services to those who are not in need in order to meet targets. Whilst health workers are likely to try and increase patient perceived quality, they may compromise on those aspects of quality that matter less to patients, in order to maximise output. There is also the risk of compromising on equity in order to achieve efficiency goals. This may occur if healthcare providers shift their focus from ‘hard to reach’ groups to more accessible and, typically, better-off groups who are more likely to seek care—‘cherry picking’ [3]. Further, providers may be better able to meet targets in less deprived areas and, therefore, be more likely to receive bonuses in these areas, potentially increasing geographical inequity in resource availability between providers [20].

There are also challenges in satisfactorily monitoring performance. Providers may be tempted to artificially inflate performance reports in order to maximise on bonuses. This practice, referred to as ‘gaming’, which has been reported in high-income settings [21], is more likely to occur when validation of performance reports is conducted internally by actors benefiting from the bonus system, and/or when transaction costs of validation are high, limiting the conduct of such activities [9,14].

### Pay for Performance (P4P) in Tanzania

The Ministry of Health and Social Welfare (MOHSW) in Tanzania with support from the Clinton Health Access Initiative (CHAI) launched a P4P programme in one region of Tanzania, Pwani, with funding from the Norwegian Ministry of Foreign Affairs in January 2011. P4P was conceived as a tool to accelerate the attainment of Millennium Development Goals (MDGs) 4 and 5. The P4P initiative is implemented in all seven districts within the Pwani region. All facilities, including hospitals, health centres and dispensaries within these districts are eligible to participate in the scheme, irrespective of ownership, on the condition that they provide reproductive and child health (RCH) services. Facilities were also required to provide full 2010 Health Management Information System (HMIS) data, and have bank accounts in order to qualify for participation. In many districts, primary level health facilities (health centres and dispensaries) do not have bank accounts, and hence, these accounts were set up as part of the programme. Stakeholders responsible for supervising facilities at district and regional levels are also eligible for bonuses (*i*.*e*., Council Health Management Teams (CHMTs) at the district level; and the Regional Health Management Team (RHMT) at the regional level).

Performance targets were based on baseline performance in the targeted areas. Targets are fixed, although their weighting in the overall performance score calculation may change as performance improves. Bonus payments are based on indicators for maternal and child health services, HMIS strengthening, facility management, and overall performance across all P4P indicators. Facility level indicators include: couple year protection rate; proportion of antenatal clients on malaria prophylaxis; proportion of newly delivered mothers who attended postnatal clinic within seven days; proportion of children under one year of age who received Penta3 and the measles vaccination; and HMIS correctly filled and submitted to the CHMT on time.

Additional performance indicators linked to bonus payments at hospitals, health centres and larger dispensaries include: percentage of HIV positive clients on anti-retroviral therapy (ART) for the prevention of mother-to-child transmission (PMTCT); percentage of newborns receiving the oral polio vaccine OPV0; and proportion of facility-based deliveries.

The proportion of correctly completed and utilized partographs is an additional indicator, for hospitals only.

The CHMTs and RHMT are assessed based on the percentage of maternal and newborn deaths appropriately audited on time, and the overall performance of the facilities in their catchment area. In addition, CHMTs are monitored on the proportion of facilities receiving quarterly district health profile reports; the proportion of facilities in the district that are included in the HMIS monthly reports; and the proportion of facilities in the district reporting stock outs of tracer medicines. The RHMT is monitored on the proportion of districts included in the HMIS reports, and submissions of semi-annual reports to the MOHSW.

Contracts are signed between facilities and their respective CHMTs, with addenda to specific targets being made every six months, based on the previous six-month performance cycle. Contracts are also signed between: the CHMT and the District Executive Director (DED); and between the RHMT and the Regional Administrative Secretary (RAS).

Facility performance reports are verified by the CHMT and the Regional Certification Committee (RCC); district performance reports are validated by the RCC, and RHMT reports are validated by the National Verification Committee (NVC), who approves payment of bonuses by the National Health Insurance Fund (NHIF) that acts as fund holder. Random checks are carried out by an independent verifier.

Payment cycles occur every six months. The first payment cycle tied to performance covered the period January to June 2011, with payment scheduled for September 2011, but actually being made in early 2012.

The bonus is paid to the facility for disbursement to staff and for facility operations. The maximum bonus per facility per cycle ranges from around $723 in dispensaries to $7,875 in hospitals. The staff top up amounts to about 10% of typical monthly salaries for maternal and child health workers.

An advisory committee was established to offer strategic direction and support to the programme and meets quarterly. The management team overseeing operations includes members of the MOHSW and of the CHAI.

This paper presents the protocol for the evaluation of the P4P programme in Pwani region, providing an overview of the framework and methods of the evaluation.

### Evaluation Framework

**Figure 1:**
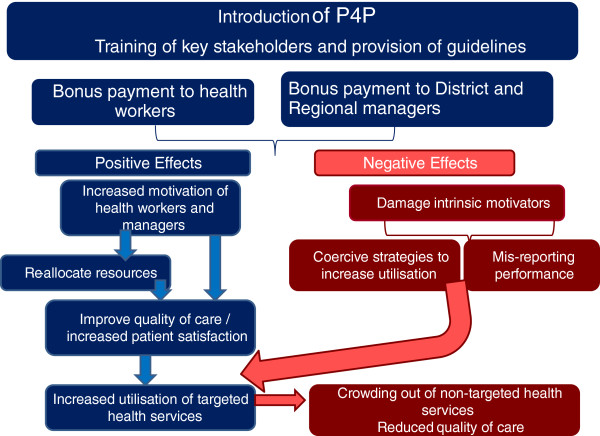
Conceptual framework.

P4P is expected to improve maternal and newborn health through a number of pathways. There are also a number of potentially unanticipated consequences (or risks) of P4P that need to be monitored in order to provide timely recommendations for improved implementation. Figure 1 presents a simplified overview of the theory of change underpinning the evaluation that was developed with reference to existing literature and based on discussion with national stakeholders.

The effect of the P4P scheme will depend on how the programme is implemented and how those stakeholders involved in or affected by the implementation react to the scheme. When fully implemented, P4P is expected to improve the quality of care of targeted services through an increase in health worker and manager motivation to obtain bonus payment, which in turn is assumed to increase service coverage among the population.

There are a variety of ways by which this could occur. All rely on health worker and manager motivation to obtain the bonus payment. Motivation will be a function of the perceived attainability of the performance targets, and the adequacy of the bonus payment level (the incentive effect) as well as potential variations in these perceptions over time. If motivated, health workers might improve the quality and accessibility of the targeted services in order to encourage patients to attend the facility by, for example, reducing waiting time, ensuring drugs are available at the facility, following clinical guidelines that may lengthen consultations, reducing user charges associated with targeted services to make them more affordable, and being more friendly and attentive to patients, resulting in greater patient satisfaction. The P4P programme may also affect intrinsic motivation. This effect may be positive or negative, depending on how the bonus scheme is perceived by health workers (as fair/unfair; as a form of recognition or as form of control) [22].

It is anticipated that regional and district managers and health facilities may adapt their methods of allocating resources in order to strengthen the quality of targeted services. In the long term, there is the possibility that the strategy may indirectly promote equity, by encouraging health workers to work in underserved facilities where there are fewer staff in order to increase the size of their bonus payment.

There are also a number of non-desired effects that could result from the P4P scheme. It is possible that the introduction of a bonus payment associated with the provision of particular health services may result in health workers adopting coercive or potentially harmful strategies to ensure sufficient numbers of patients attend facilities, such as delaying or avoiding referral of complicated pregnancies in order to increase the number of deliveries at the facility. It is also possible that facilities misreport target indicators, inflating numbers in order to benefit from the bonus. While this should, in principle, be detected by the district and regional levels during data validation, there could be a potential conflict of interest as these stakeholders also benefit from a bonus payment, which might allow for such distortions to occur more readily. There is also the risk that the utilisation and quality of health services that are not included in the bonus payment will reduce, due to ‘crowding out’ by targeted services. Furthermore, some aspects of quality of the targeted services may decline over time, if health workers become overburdened and utilisation increases beyond available facility capacity.

### Objectives of the evaluation

The objectives of the evaluation are:

1. To assess the effect of the P4P initiative on the quality and coverage of targeted maternal and newborn healthcare services and selected non-targeted services at facilities.

2. To examine the implementation process, stakeholder response to and acceptability of the P4P design and potential unintended consequences.

3. To measure the cost-effectiveness of the P4P programme.

To address these objectives, there are three components to the evaluation: an impact evaluation, a process evaluation, and an economic evaluation. The specific objectives and methods of each component of the study are reviewed in turn.

## Methods

### Impact evaluation methods

#### Study design

The impact evaluation will employ a controlled before and after study design. Surveys will be undertaken within all seven districts in Pwani region where P4P is being implemented before and after its introduction and also among four control districts with no P4P, namely: Kilwa, Mvomero, Morogoro town, and Morogoro rural. Control districts were selected from two neighboring regions such that they were as similar as possible to intervention districts in relation to poverty and literacy rates, the rate of institutional deliveries, infant mortality, population per health facility, and the number of children under one year of age per capita. Care was also taken to avoid districts where programmes were underway to improve maternal and child health, which could confound results.

**Figure 2:**
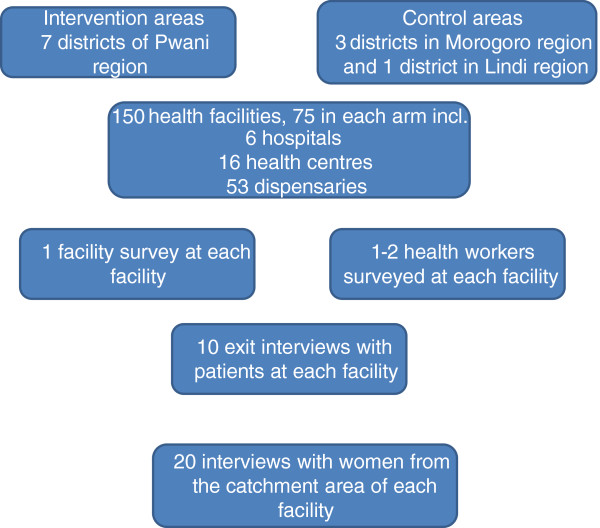
Overview of impact evaluation data collection tools and sampling strategy.

The impact evaluation relies on four tools that will be administered at baseline and endline: a health facility survey, a health worker survey, a survey of patients exiting facilities, and a survey of women who delivered in the previous 12 months (Figure 2). The facility survey, health worker survey, and exit interviews will be conducted at all sampled facilities. The household survey will be administered to households within the catchment areas of these facilities to complement the data compiled during the facility survey [11].

### Data collection tools

The health facility survey aims to measure the effects of P4P on service availability and provision at the sampled facilities. It is comprised of three sections. In the first section, questions focus on basic service provision within the facility (staffing levels, opening hours, facility management, as well as facility infrastructure). The second section of the survey compiles equipment and drug availability data. The third section captures HMIS data on service utilisation, facility expenditures and revenues for the 12-month period before P4P was implemented (January to December 2010 (at baseline) and the period from January 2011 to December 2012 (at endline). The health facility survey will be administered to the facility in-charge or in his/her absence to a knowledgeable health worker or administrator.

The health worker survey tool aims to measure the effects of P4P on health workers’ working conditions and attitudes towards work at the selected facilities.

The exit interview survey primarily intends to measure the effect of the P4P initiative on a range of subjective and objective indicators of quality of care for targeted and selected non-targeted services. The survey will also examine the effect of P4P on the cost of these services. Respondents eligible for interview include women of reproductive age (aged between 16 to 49 years) attending antenatal or postnatal care, or women with children under-one year of age coming for a preventive check up or an immunisation for the baby. These patients will respond to questions linked to the services targeted by P4P. Patients attending care for non-targeted services will also be interviewed. Non-targeted service users will include: women of reproductive age who are not pregnant, or children under five years of age accompanied by a woman of reproductive age, reporting with fever and no cough (as a proxy for malaria), or fever and cough (as a proxy for acute respiratory infection – ARI), or diarrhoea. These conditions were chosen as they were the three most significant conditions reported at outpatient departments in Tanzania in 2009.

A survey of women who had delivered within the previous 12 months will also be carried out. The women’s survey addresses the effects of P4P on service use during pregnancy, place of delivery, birth weight and postpartum care and care for the newborn as well as related costs and service satisfaction. Household socioeconomic status is also measured in this survey. The core indicators for each of the surveys are shown in Table 1.

**Table 1:** Overview of core indicators for each of the surveys

**Type of impact**	**Indicators**	**Data source**
Quality of care	% babies weighed at birth	Women’s survey
	% patients prescribed drugs outside facility	Exit interview
	Average waiting time for targeted services in minutes	
	Average waiting time non-targeted services in mins	
	Average consultation time for targeted services in mins	
	Average consultation time for non targeted services in mins	
	% reporting overall quality satisfactory	
	% did blood test during ANC	
	% did blood pressure during ANC	
	% prescribed iron tablets during ANC	
	% prescribed drugs for malaria during ANC	
	% counselling for HIV	
	% tested for HIV	
	% women examined during PNC	
	% health workers satisfied with medicine availability	Health worker survey
	% health workers satisfied with functioning equipment availability	
	% health workers satisfied with medical supplies availability	
	Mean no. Of clinical cadre	Facility survey
	Mean no. Of nursing cadre	
	Mean no. Of paramedical cadre	
	% facilities being renovated in past year	
	% facilities with access to electricity	
	% facilities with access to piped water/hand pump	
	% facilities with toilet facilities	
	% facilities offering 24 hour delivery services	
	% facilities where skilled providers attend home deliveries	
	Average no of beds in the maternity ward for health centers/hospitals	
	% facility with stock out of DPT vaccine type in past 90 days	
	% facility with stock out of measles vaccine in past 90 days	
	% facilities with oxytocin stock outs in past 90 days	
	% facilities with ORS stock outs in past 90 days	
	% facilities with stock outs of all ARVs in past 90 days	
	% facilities with partograph stock outs in past 90 days	
	% facilities with gas for vaccine refrigeration stock outs in past 90 days	
	% facilities reporting all contraceptive pill types stock out in past 90 days	
	% facilities reporting delivery kits stock out in past 90 days	
	% facilities reporting broken equipment disrupted the provision of services in past 90 days	
Service utilization	% women delivering in a health facility	Women’s survey
	% of women who had 4 or more ANC visits	
	Average months pregnant at first ANC visit	
	% c-section rate	
	% newborn immunised before going home	
	% women treated for HIV	Women’s survey and exit interview
	% women who received postnatal care within 2 months of birth in a health facility	
	Number of PNC visits in a health facility	
	% of women who were examined during PNC	
	Timing after birth for first visit in days	Women’s survey
	% of children getting BCG	
	% of children fully immunised for polio (among appropriate age group)	
	% of children fully immunised for DPT (among appropriate age group)	
	% measles fully immunised for measles (among appropriate age group)	
	% women currently using a family planning method	
	Mean annual outpatient visits under 5	Facility survey
	Mean annual outpatient visits all age groups	
	Mean annual inpatient admissions under 5	
	Mean annual inpatient admissions all age groups	
	Mean annual ANC service utilization (all ANC and first ANC)	
	Mean annual delivery service utilization (normal delivery)	
	Mean annual FP visits	
	Mean number of under 1 year olds receiving DPT vaccine	
	Mean number of under 1 year olds receiving polio vaccine	
	Mean number of under 1 year olds receiving measles vaccine	
	Mean number of infants receiving BCG vaccine	
Motivation, work conditions	% health workers reporting increase in working hours in past 12 months	Health worker survey
	% health workers reporting last external supervision occurred in past 90 days	
	% health workers receiving salary increase in last 12 months	
	% health workers reporting performance to be the reason for salary increase	
	% health workers reporting being motivated to work hard	
	% health workers satisfied with their salary	
	% health workers satisfied with their employment benefits	
	% health workers satisfied with promotion opportunities	
Economic effects	% paying for delivery at public facility	Women’s survey
	% patients attending targeted services paying for services	Exit interview
	% patients attending non-targeted services paying for services	
Equity	% service use among poorest compared to least poor women/children	Women’s survey and exit interview
	% reporting payment for services among poorest compared to least poor women	
Health - behavioural	Average weight of baby in kg	Women’s survey
	% reporting small baby	
	% breastfeeding within 1 hr of birth	
	Mean annual number of low birth weight babies	Facility survey

### Sampling

The health facility is the primary sampling unit. Facilities were sampled from those that were eligible to participate in the P4P scheme (they offered reproductive and child health services and had submitted a one year backlog of HMIS data, enabling performance targets to be measured). All eligible hospitals (n = 6) and health centres (n = 16) from the intervention districts were included in the sample along with all eligible non-public dispensaries (n = 11). An equivalent number of facilities in control areas were sampled by level of care. Public dispensaries were sampled at random with probability proportional to the number of public dispensaries in a given district (n = 42). In control areas, hospitals and health centres were sampled to match as closely as possible with selected intervention facilities in terms of annual outpatient care visits and staffing levels. A total of 75 health facilities were sampled from intervention districts, and 75 were sampled from control districts (Figure 2). In Pwani region, 46% of all facilities in the region were included in the sample.

The aim of the sampling procedure for the selection of health facilities was to seek district representation, while for the health worker survey it was to obtain the views, attitudes, and perceptions of at least one health worker per facility. No sample size calculation was therefore carried out. In dispensaries, one health worker will be interviewed. If more than one health worker is on duty, preference will be given to someone other than the in-charge to avoid overburdening them with questions (as they will be interviewed for the facility survey). In health centres and hospitals, two health workers will be interviewed. The health workers will be selected at random from those who are on duty at the facility on the day the interviewers are present.

For the exit and household surveys, the sample size calculation was based on the formula by Hayes and Bennett, 1999, adjusted for the cluster design of the study at the facility level [23]. We estimated the size needed to detect a 17% reduction in waiting time from 114 minutes (SD 66) [24] to 95 minutes, with a k value of 0.25, 80% power and a significance level at of 5% (two tailed test). We did not increase the sample size to account for non-response because response rates of 100% were observed in previous studies in Tanzania [25,26]. The estimated sample size was 10 exit interviews per facility, equivalent to a total of 750 interviews in intervention and control areas respectively. A balance in the number of interviews between antenatal, postnatal clients and non-targeted services will be sought.

Exit interview patients will be approached by interviewers upon entry to the health facility and asked a series of screening questions to check their eligibility. Eligible patients will then be asked for their informed consent to participate in the study. This process will be repeated until the required number of eligible consenting respondents has been attained. Participants will then be monitored by the interviewers from their time of arrival at the facility until their time of departure, and the waiting and consultation times will be measured using a stopwatch. The cadre of the provider seen by the woman/child will also be recorded by the interviewer. The survey tool will be administered to patients upon completion of their consultation in a quiet location within the facility, at distance from providers and other patients.

For the household survey, we estimated that the required sample size to detect an 11 percentage point increase in institutional deliveries (from 50 to 61%), with k value of 0.25, 90% power, and a significance level at of 5% (two tailed test), and a 90% response rate, was 20 households per cluster, equivalent to 1,500 women per study arm. The following process was followed to identify eligible households. First, villages were sampled from the facility catchment area; for all dispensaries, the village where the facility is located will be selected by the research team; for health centres and hospitals, two villages will be selected at random from all villages lying within the ward where the facility is located. Second, all hamlets (comprising approximately 100 households) within this village/these villages, and located within the catchment area of the facility will be identified; a random sample of four of these hamlets will then be selected. In the case of dispensaries, all four hamlets will reside within the selected village. In the case of health centres and hospitals, two hamlets will be sampled from each village. Third, five households will be sampled from each of the selected hamlets, amounting to a total of 20 households within each facility’s catchment area; households will be selected at random from the selected hamlets using a modified Expanded Programme of Immunisation (EPI) type sampling scheme that ensures an equal chance of any household being selected.^1^

### Process evaluation

The process evaluation aims to compare what was planned to what is actually happening in practice, incorporating issues related to the acceptability of the scheme to various stakeholders, as well as the context of implementation, in an attempt to understand potential facilitating or debilitating factors that might explain variation in implementation. We will track indicators of health worker and manager satisfaction, supervision and verification, and implementation constraints and facilitators; as well as examine how specific issues such as facility ownership, facility resources, and governance structures affect the implementation of the programme at the facility level. The process data will be used to get a better understanding of the reasons for any P4P effect or a lack of effect, as well as potential unintended consequences. Table 2 presents a list of qualitative indicators used to monitor the progress of implementation at district level.

**Table 2:** Process monitoring subthemes

**Specific objective**	**Qualitative indicators**
Acceptability of P4P	- Size of bonus payment and fairness, and rationale (support or control).
	- Extent to which targets are achievable.
	- Effect of P4P on working relations between stakeholders
	- Difficulties/ ease of implementing P4P
	- Extent to which bonus payments are motivating, and changes in perceptions over time
Context of implementation	- District leadership and governance of health services
	- Supervision
	- Access barriers to care (geographical, transport, cultural; other)
	- Other interventions underway that target maternal and child health and may influence observed outcomes;
	- Other changes in society that may affect access and utilization of health services.
Implementation process	- Training process
	- Awareness and understanding of P4P
	- Availability and use of guidelines and/or operating procedures, ease of following
	- Contracting process
	- Reporting process
	- Bank accounts
	- Bonus payment transparency
	- Timeliness of bonus payments
	- Methods for dealing with misreported indicators
	- Use of bonus payments - adherence to guidelines, transparency, fairness?
System changes	- Effect on procedures and practices of recording and reporting of information.
	- Fund management
	- Supervision frequency and content

Three rounds of qualitative data will be collected throughout the programme life cycle to explore how perspectives and knowledge change over the course of implementation and to inform the continued implementation of the programme.

The first round of data collection will take place in a sample of 15 health facilities from five of the seven intervention districts. A total of 54 interviews and four focus group discussions (FGDs) will be conducted with key stakeholders at facility, district, regional, and national levels. Districts were selected to offer variation in relation to geographical location, skilled birth attendance coverage and achieved performance targets during the first cycle. Three facilities were purposively selected within each district to offer a mix of ownership and level of care as well as differing levels of baseline performance.

In rounds two and three, data will be collected from facilities in a subsample of the round one districts using a mix of individual interviews and focus group discussions.

Interview guides for each round were developed for the following stakeholder groups (the CHMTs, the district P4P focal person, health facility in-charges, health workers, the RHMT, the Health Facility Governing Committee (HFGC), and national level stakeholders). Contextual information will also be collected in each district.

### Economic evaluation

The objective of the economic evaluation is to ascertain whether P4P represents value for money. The study will be carried out from a societal perspective, which includes all agencies or bodies that are involved in implementation or who incur costs or may be affected by the intervention, for example: the implementers (*e*.*g*., government (MOHSW, the Prime Minister’s Office – Regional Administration and Local Government (PMO-RALG)), the NHIF, and CHAI; as well as those who are affected by implementation and may incur costs as a result (*e*.*g*., households, health workers, district health managers).

We will estimate both the financial costs of each activity (*i*.*e*., what is paid out by the funding body—all financial transactions), as well as the economic costs, which include the value of all resources valued at their opportunity cost. Similarly, any donated or subsidised items will be valued at market prices.

Costs and cost-effectiveness of the P4P programme will be compared to the current situation (doing nothing). Costs will be classified according to project activities (start-up activities and ongoing activities) as well as by resource inputs (recurrent items such as staff, supplies, transport etc., as well as capital costs such as equipment, vehicle etc.). Capital items will be annualised over the lifetime of the project. Start-up costs will include those resources used during training of providers and other stakeholders; contracting; entering baseline HMIS data, and target setting; provision of guidelines; and establishment of a steering committee. Ongoing costs will include those resources used during data processing; verification; strategies adopted by facilities, districts, and regions to meet targets; fund payout; and review and modification of targets.

Programme cost data will be derived from project accounts and through interviews with key implementation stakeholders. Interviews with district, regional, and national stakeholders will be undertaken to allocate staff time to activities as well as to identify and value resources and time invested in the programme that is not paid for. Facility data will be used to ascertain to what extent increased service use results in costs to the health system in terms of additional staff or beds, for example, or to what extent there is spare capacity and such an increase can be readily absorbed within the system. Household costs will be captured during the baseline and endline household surveys and will enable the measurement of the societal costs of an eventual service increase due to the programme, as well as changes in the levels of out of pocket payments.

### Data management

To provide quality assurance within the impact evaluation, survey data will be checked by supervisors at the end of each day of data collection. Household, exit, and health worker interview data will be collected using hand held devices (Samsung Galaxy tablets 7.0 and Huawei IDEOS phones) with skip and quality check functions to minimize data entry error. Facility data will be captured on paper and double entered. Data will be backed up on CD each day in a Microsoft Access Database, and converted to Stata for analysis. Hard copies of questionnaires will be stored in a lockable room. Electronic output will be anonymised.

Interviews and focus groups conducted as part of the process and economic evaluation will be conducted in Kiswahili and recorded using sound digital recorders. Audio sound files will be transcribed and translated into English by the bilingual researchers who conducted the interviews. All translated data obtained from interviews and focus group discussions will be entered into QSR Nvivo 9 for data management, for the process evaluation, and into Microsoft Excel for the economic evaluation.

### Analysis strategies – quantitative data

Impact data will be checked first for consistency and after export to Stata, data cleaning will be undertaken. Binary variables (Yes = 1, No = 0) will be created for all categorical variables. All binary and continuous variables will be summarized by calculating means and standard deviations. A comparison of all variables between intervention and control arms will be made at baseline. Tests of differences in means between intervention and control groups will be conducted using the Adjusted Wald F-test. Principal component analysis (PCA) will be used for creating socioeconomic status (SES) indices for household and exit interview data analysis using data collected on household size and characteristics, access to utilities, durable asset ownership, food security, household expenditures, head of household marital status, highest level of education attained, and main occupation. Data that use a Likert scale (*e*.*g*., dissatisfied = 1, neither satisfied nor dissatisfied = 2, satisfied = 3) will be analyzed by calculating individual mean scores for each variable. Factor analysis will be used on patient satisfaction and health worker motivation data for identifying the underlying factors or themes within the data.

At endline, we will compare the main outcome indicators for each of the survey tools between intervention and control arms, using data for twelve months of intervention. We will estimate a multivariate regression specification of the difference – in - difference model in which an individual’s (woman, patient, health worker, facility) outcome is regressed against a dummy variable, indicating whether the facility was eligible for P4P (*i*.*e*., providing reproductive and child health services, had submitted the backlog of HMIS data, and performance reports), a facility fixed effect, a year indicator, and a series of individual and household characteristics (in the case of patients and women). For household and exit data, we will calculate robust standard errors, clustered at the facility level to correct for correlation of the error terms across patients within facilities, and across households in facility catchment areas.

### Analysis strategies – qualitative data

Transcripts from interviews and focus group discussions will be read systematically and independently by each of the researchers, and coded applying thematic content analysis, which identifies recurrent themes that form a cluster of linked categories containing similar meanings. To validate findings, we will triangulate data across respondent groups and look for supporting documentary evidence, where available. Analyses will be undertaken on an ongoing basis as transcripts and other information from the study sites become available.

### Ethical issues

The evaluation study was approved by the Institutional Review Board of the Ifakara Health Institute and the Ethics Review Board of the London School of Hygiene & Tropical Medicine. The study design and protocol were also approved by the P4P Management Team that includes members of the MOHSW. Presentations of the proposed research methods were also made to the P4P steering committee that includes CHAI, MOHSW and the Government of Norway. Prior to undertaking data collection, letters were sent to respective District Executive Directors (DEDs) copied to District Medical Officers (DMOs) informing them of the study and its objectives. Subsequently, visits were made to the DMOs to agree on dates for data collection. An information sheet was left at the DMO’s office. Information sheets and consent forms were provided to all those participating in the study. Written consent was obtained prior to undertaking all interviews and FGDs.

## Discussion

The introduction of P4P in Tanzania aims to increase service utilisation, and also stimulate better quality of care, by making the system more responsive to user needs. However, health worker response to financial incentives, and potential for improving quality of care and service uptake, in a context where system constraints are substantial, and supervision systems are weak, is unclear.

This evaluation will contribute robust evidence on the impact and cost-effectiveness of P4P in a low-income setting, as well as generate a better understanding of how complex intervention packages like P4P interact with the health system. This will be the first evaluation study of a P4P scheme that examines the intervention impact, its cost-effectiveness, and process of implementation. This will contribute to an expanding knowledge base in this area, and address questions that have not previously been explored in low-income settings, such as the cost-effectiveness of P4P, and its impact on equity: do better off households benefit more from service access?; are better-off facilities better able to achieve targets, and access bonus payments? The study will provide a close examination of the process of implementation and contribute to understanding the factors associated with successful performance and some of the barriers to achieving targets, potential unintended consequences, as well as the effect on service use and quality of targeted and non-targeted services.

The main design constraint facing the evaluation is the timing of the baseline evaluation, which took place during the course of the first payment cycle but before payments were made in most cases. The risk is that baseline data will already be affected by the intervention, minimising the overall observed P4P effect. Process data revealed that knowledge of the programme was very limited at this time. To get around this issue in the household survey, households with women who had given birth prior to the first payment cycle will be selected. Health workers will be also asked to report on their working conditions and practices prior the introduction of P4P. The facility survey will compile information on the situation in the past 90 days, and service use statistics in the previous year (at baseline) and two years (at endline). The survey tool that is most susceptible to the timing effect is the exit interview. If baseline differences are identified between intervention and control sites, that lend support to a potential P4P effect, then appropriate adjustments will be made in the endline analysis.

A further limitation is the short time frame for the impact evaluation that evaluates effects over a one year period. The process evaluation explores changes over the time frame of the evaluation but it will not shed light on the extent to which these changes are sustained in the longer run.

## Endnote

^1^At the centre of the hamlet the supervisor throws a pen to determine the direction, and counts 10 houses in the direction indicated by the pen. A number is picked at random by writing down ten numbers and picking one at random. The house with the corresponding number is the starting point for data collection. The supervisor introduces the study to the household head, or a representative of the household and asks him/her if there are any eligible women living in the household: a woman aged 16 to 49 who had a baby between Oct 2010 and Oct 2011. If there is an eligible woman, they leave a copy of the consent form with them and ask if it would be convenient to return for interview the next day, at an agreed time. The pen is then thrown again and the next household in the direction of the pen is selected for interview. The supervisor continues going household to household in this way until five eligible households consenting to being interviewed are identified. If there is a junction in the path, the supervisor throws a pen again to determine the direction.

## Abbreviations

ART: Anti-retroviral therapy; CHAI: Clinton Health Access Initiative; CHMT: Council Health Management Team; DED: District Executive Director; DMO: District Medical Officer; EPI: Expanded Programme of Immunisation; FGD: Focus Group Discussion; HFGC: Health Facility Governing Committee; HMIS: Health Management Information System; P4P: Pay for Performance; MDG: Millennium Development Goal; MOHSW: Ministry of Health and Social Welfare; NHIF: National Health Insurance Fund; NVC: National Verification Committee; PCA: Principal component analysis; PMO-RALG: Prime Minister’s Office – Regional Administration and Local Government; PMTCT: Prevention of Mother to Child Transmission; RAS: Regional Administrative Secretary; RCC: Regional Certification Committee; RCH: Reproductive and child health; RHMT: Regional Health Management Team; SES: Socio-economic status.

## Competing interests

The evaluation study was funded by the Norwegian Ministry of Foreign Affairs who also funded the P4P programme in Pwani region. However, authors declare that the evaluation is being carried out independently of the programme’s implementation and that they have no competing interest.

## Authors’ contributions

JB, PJ, and EP developed the impact and economic evaluation protocols, MM, IM, IM, and IN developed the process evaluation protocol. JB, MM, OM, and SA developed the initial grant application proposal. All authors contributed to the current manuscript. All authors read and approved the final manuscript.

## Authors’ information

JB is a Senior Lecturer at the London School of Hygiene & Tropical Medicine, who was seconded to the Ifakara Health Institute from 2007–2012; and is a co-Principal Investigator (PI), leading the impact and economic evaluations. PJ is a Research Scientist at the Ifakara Health Institute; researcher on the impact and economic evaluation. IM is a Research Scientist at the Ifakara Health Institute; researcher on the process evaluation. IM is a Research Scientist at the Ifakara Health Institute; researcher on the process evaluation. IN is a Research Officer at the Ifakara Health Institute; researcher on the process evaluation. MM is a Senior Research scientist at the Ifakara Health Institute; and is a co-PI leading the process evaluation. EP is a Lecturer at the London School of Hygiene & Tropical Medicine and has been seconded to the Ifakara Health Institute since 2012; technically supporting the impact and economic evaluations. SA is the Director of the Ifakara Health Institute; and is the overall PI of the P4P evaluation study. OM is the Director of the CMI lending technical support to the P4P evaluation.
